# Counting colonies of clonogenic assays by using densitometric software

**DOI:** 10.1186/1748-717X-2-4

**Published:** 2007-01-09

**Authors:** Maximilian Niyazi, Ismat Niyazi, Claus Belka

**Affiliations:** 1 CCC Tübingen, Department of Radiation Oncology, Hoppe-Seyler-Str. 3, 72076 Tübingen, Germany; 2 Bureau for Technique and Documentation, Paracelsusstr. 21, 70599 Stuttgart, Germany

## Abstract

Clonogenic assays are a useful tool to test whether a given cancer therapy can reduce the clonogenic survival of tumour cells. A colony is defined as a cluster of at least 50 cells which can often only be determined microscopically. The process of counting colonies is very extensive work and so we developed software that is able to count the colonies automatically from scanned flasks. This software is made freely available by us with a detailed description how to use and install the necessary features.

## Background

Everyone who has already counted colonies from a clonogenic assay knows that this is hard work which takes a lot of time. Many groups have thought about an improvement of the counting system [1-8] and the cited publications certainly won't cover all attempts, but no method has achieved a widespread use at all.

One may ask what the reasons are. Two major reasons are certainly the following: all presented methods had problems when faced with clustered colonies. Some systems have managed to avoid this problem by constructing ingenious scanning systems, but these systems are only commercially available.

For this purpose we developed a new software that is able to evaluate the clonogenic assays by using densitometric methods and other special parameters. This revealed highly fitting results which were compared to manual countings with the microscope.

## Procedure and results

The newly designed software requires normally scanned six-well-plates or flasks (200 dpi is optimal for excellent results) which should be scanned as grey-scaled photos and saved as jpeg-file. Even better is a reflection light scanner but this is no condition for good results. It is anyhow recommended to avoid (as much as possible) disturbing shades which are caused by scanners, although the software can usually recognize and neglect these shades.

Flasks can be scanned and evaluated without editing the scanned pictures. In our institute it is a routine activity to archive the clonogenic assays by scanning them so that this is no additional work.

**Figure 1:**
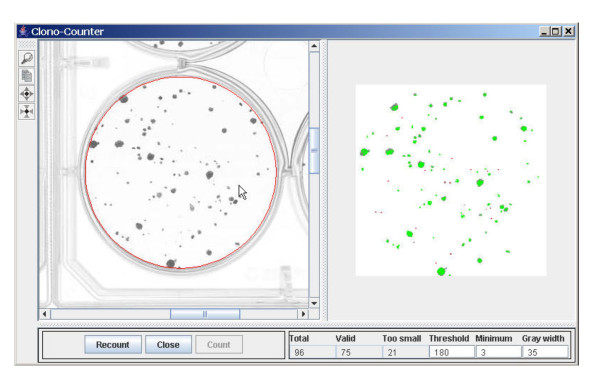
**Screenshot of the program**. Left panel: the scanned flask, the circle marks the region that will be counted; right panel the result: green areas denote a colony, red points mark the clusters which are too small. Parameters and counted points are displayed below.

When launching the program (a more detailed description is given in the manual of the program, see additional file 1) you are asked to select a data file. After selecting a file the program will start (see fig. 1). The area to be counted can be selected and copied to the working area. By counting the first time you usually won't get the result you expected. So you have to adjust the available parameters. This presumes that you have counted one well of the plate (usually the one that contains the minimum amount of colonies). In our experiments we had several dilutions of cells with the same treatment; so for each treatment we counted one well with around 20 cells and could adjust the parameters for the program.

The software provides three independent parameters: the gray level, the maximum size of one colony (defined by the area) and another parameter that considers the distribution of the gray colour within the colony. This is done by writing every cluster of the scanned area into several matrices which contain information on localisation and gray level.

One needs some experience to find the right parameters, but with the help of some tricks (see the manual) you can manage it in a short time.

But it is not only the match of the counted numbers which gives evidence on the right parameters. The program displays which colonies were counted and which not. This allows a manual correction with respect to the golden standard (the microscope).

We compared this attempt with the microscopical evaluation and found a relative mistake < 5%.

## Discussion

We developed a new system for automated counting of colonies on cell culture flasks that uses an algorithm that has not been used before.

This attempt requires three parameters which are determined by comparison to the microscopical evaluation of single wells which leads to high economy of time.

The results showed excellent matching (relative mistake < 5%) as we compared that to manual counting (dependent on the person, but normally 5 – 10%) or to the golden standard (microscope) which has after all an interindividual difference of around 2%. Another argument is the fact that this relative mistake is made for all wells and as (for a clonogenic assay) the plating efficiency is considered, this does not play a key role according to the overall result.

The program is written in Java and can be downloaded. It is freely available and we are interested in feedback and whether you can use the program for your practical work. Detailed advice for installation and operation is given in the manual.

One may ask why one has to make new adjustments for every treatment. For similar treatments this is in fact not necessary. But some cell lines change their shape following very different treatments. Sometimes cells grow under treatment and so one has to set another cut off for the minimum size compared to the control.

But this did not influence the inherent advantage of automated counting according to economy of time.

## Supplementary Material

Additional File 1 Clono-Counter. Contains a manual for the program, the program itself and an example. Click here for file
