# Supplementary material for: Counting colonies of clonogenic assays by using densitometric software
Source: Radiat Oncol. 2007 Jan 9;2:4. doi: 10.1186/1748-717X-2-4 (PMC1770926; doi:10.1186/1748-717X-2-4)
Supplement: Additional File 1 — Clono-Counter. Contains a manual for the program, the program itself and an example. [file 1748-717X-2-4-S1.zip › manual_clono.pdf]

Clono-Counter

Attention: Although we carefully tried to optimize the program, we are not able to adopt any responsabilities for results that were obtained with it.

Overview

Clonogenic assays are a useful tool to test whether a given cancer therapy can reduce the clonogenic survival of tumour cells. A colony is defined as a cluster of at least 50 cells which can often only be determined microscopically. The process of counting colonies is very extensive work. Several groups have developed automated counting systems, but these systems are only commercially available.

Clono-Counter instead is a small and simple program that can be used stand-alone with a clear user interface and an easy handling. But the advantages of the program are mainly localized within the internal algorithm that is able to differentiate between different colonies which are clustered together and differ just by a minimal shading difference.

How to install Clono-Counter

You have to download the J2SE Runtime Environment (JRE) from the Java-Sun-homepage:

http://java.sun.com/

Choose “Java SE” from the “Downloads” menu where you can download the latest version of the JRE and then install it on your PC. This page includes also information about JRE and how to install it.

The JRE provides the libraries, Java virtual machine, and other components necessary for you to run applets and applications written in the Java programming language.

The Clono-Counter is delivered as a zip-file. Save this file to any directory where you want to install Clono-Counter. When extracting the data an “images” folder is created. To launch Clono-Counter doubleclick the jar-file "clono.jar".

Hint: It is recommended but not necessary to save the pictures to be analyzed in the same directory where clono.jar is located. One test picture is included in the zip-file.

Preparation of pictures

Clono-Counter requires normally scanned six-well-plates or flasks (200 dpi is optimal for excellent results) which should be scanned as gray-scaled photos and saved as jpeg-files. Even better is a reflection light scanner but this is no condition for good results. It is anyhow recommended to avoid (as much as possible) disturbing shades which are caused by scanners, although the software can usually recognize and neglect these shades when using adequate parameters.

Flasks can be scanned and evaluated without editing the scanned pictures. In our institute it is a routine activity to archive the clonogenic assays by scanning them so that this is no additional work.

As we are mainly working with six-well-plates, it is possible (and useful) to scan four plates simultaneously and save them in one jpeg-file.

Work with Clono-Counter

Start the program

Immediately after launching the program the dialog box “Open” is shown. Choose a data file of the scanned picture and click “Open”.

Optimal magnification

Before starting to count you have to decide whether you want to zoom into the image or not. For this purpose you can click on to zoom into 120%. This step can be done one time and is irreversible. Once you chose this, you should perform the other countings with the same magnification as the parameters yield other results with a new magnification. You can launch the program again to open the original image.

When working with Microsoft Windows® you can use the magnifying glass which can be found at the accessories (start / programs / accessories / …). This allows an optimal assessment of the chosen parameters.

Select the area to be counted

The program counts circular selected areas. Deviations from this form can be adjusted by several correction mechanisms. Before you select a specified area all buttons are inactive except the "close" and zoom buttons.

Select a circular area: left click on the upper/left point (1) of the area to be selected, holding the mouse button clicked move the mouse pointer to the lower/right point and release the mouse button. Notice: you can select only one area, if you select another area, by repeating the steps given before, the old selection will be unselected automatically.

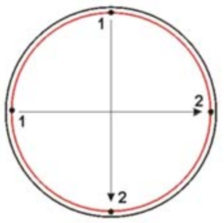

Hint: Choose an area that is a bit smaller than the specified area because the adjustment can be done afterwards.

Adjust the selection: You can change the selection in the following way:

- Click to enlarge the circular area
- Click to reduce the circular area

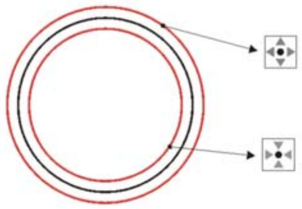

- Enlarge a sector of the circle (left / right / upper / lower sector): hold down the "Shift" key and click one of the arrows ← / → / ↑ / ↓
- Reduce a sector of the circle (left / right / upper / lower sector): hold down the "Ctrl" key and click one of the arrows ← / → / ↑ / ↓

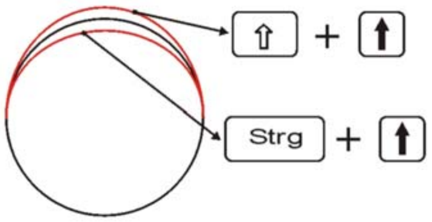

Hint: Avoid selecting the limiting branch of one circular well. This can cause shadows which would disturb a proper counting.

Copy the selected area

After selecting the right area, click on to copy the specified area into the working panel.

Counting

Now press “Count”. The program sets the parameters on certain values which (in most cases) do not yield an acceptable result. So one has to adjust the parameters after the first counting. A result is shown in the figure below. Green and simply connected areas mark one colony. The left panel shows the situation at the beginning, the right panel the counted area in the working panel. Red marked areas are not counted because they are too small. This allows controlling the specification of the parameters.

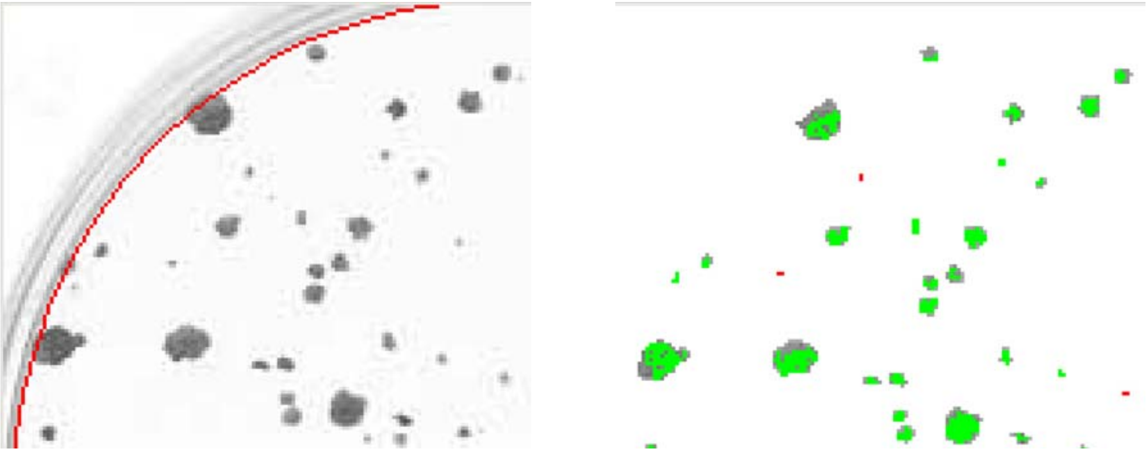

Left: original image, right: counted colonies are green-coloured, red dots will be neglected

Remark: You have to adjust the available parameters. This presumes that you have counted one well of the plate (usually the one that contains the minimum amount of colonies). In our experiments we had several dilutions of cells with the same treatment; so for each treatment we counted one well with around 20 cells) and could adjust the parameters for the program.

Repeat your counting / adjusting the parameters

In order to explain the meaning of the single parameters, we will introduce them by some examples.

Threshold

The grayscale reaches from 0 (which relates to “black”) to 255 (“white”). The background of the image is usually not white, with a gray level less than 255 and the colonies to be counted are much darker than this background and the rest of the specified area. The threshold defines the gray level of the background and in general the gray level of the area to be neglected during the counting. The figure below shows the impact if the threshold is reduced from 180 to 130. In the left figure all the gray levels between 180 and 255 will be neglected, in the right figure from 130 to 255.

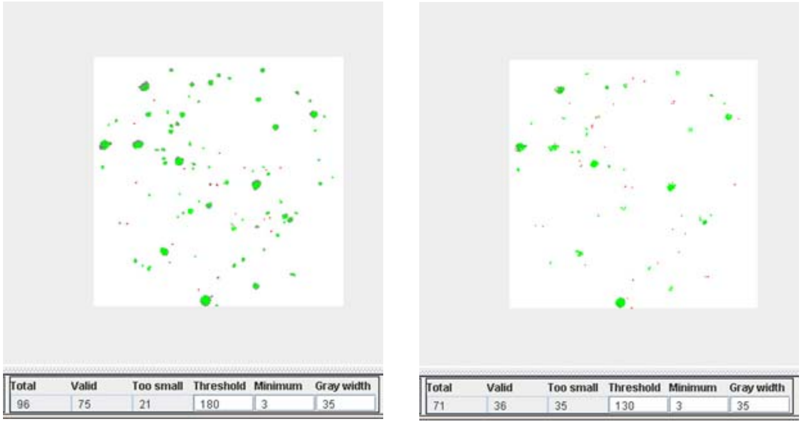

The impact of the parameter “threshold”

Minimum

The minimum defines how many pixels have to be within one green dot that it is seen as a colony. When choosing a higher value you normally get a lower number of colonies because many of the dots which were seen as colonies before, have become too small. These will appear red-coloured.

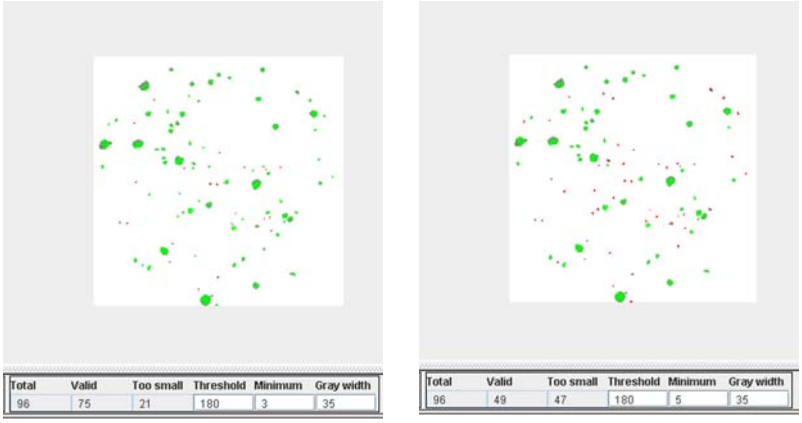

The impact of the parameter “minimum”

Gray width

The gray width is probably the most complicated part within the program. It tries to imitate the human eye which is able to recognize single colonies from clustered colonies. The gray width defines which gray levels are considered to be part of one colony. This is especially useful if two colonies with different gray shapes overlap. Then it is normal that the colours in the center are darker than in the surrounding area. Dark points are first realized by the program (as it looks in succession after points with a gray value 0, 1, …up to the threshold value), afterwards the neighbourhood of these points is analyzed. The gray width is the maximum allowed difference between the gray shades belonging to one colony. Setting a lower gray width mainly results in smaller colony size and allows an easier differentiation between clustered colonies (see Fig. below).

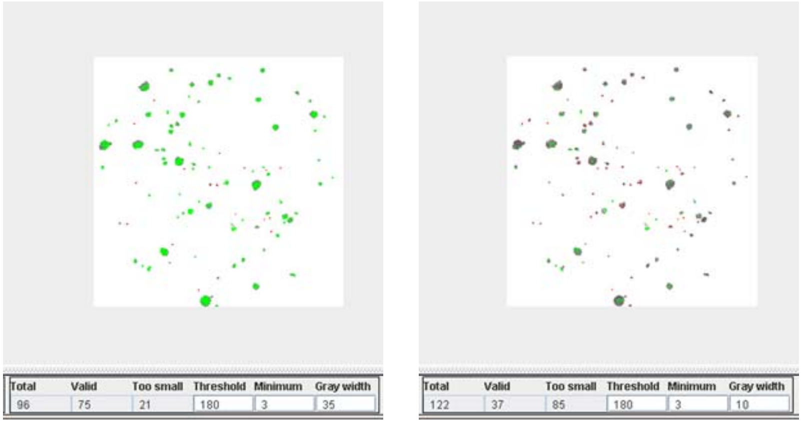

The impact of the parameter “gray width”

Discussion

We developed a new system for automated counting of colonies on cell culture flasks that uses an algorithm that has not been used before.

This attempt requires three parameters which are determined by comparison to the microscopical evaluation of single wells which leads to high economy of time.

The results showed excellent matching (relative mistake < 5 %) as we compared them to manual counting (dependent on the person, but normally 5 - 10 %) or to the golden standard (microscope) which has after all an interindividual difference of around 2 %. Another argument is the fact that this relative mistake is made for all wells and as (for a clonogenic assay) the plating efficiency is considered, this does not play a key role according to the overall result.

The program is written in Java and can be downloaded. It is freely available and we are interested on feedback and whether you can use the program for your practical work. Detailed advice for installation and operation is given in the manual.

One may ask why one has to make new adjustments for every treatment. For similar treatments this is in fact not necessary. But some cell lines change their shape following very different treatments. Sometimes cells grow under treatment and so one has to set another cut off for the minimum size compared to the control.

But this did not influence the inherent advantage of automated counting according to economy of time.
