# Supplementary figures and images for: Counting colonies of clonogenic assays by using densitometric software
Source: Radiat Oncol. 2007 Jan 9;2:4. doi: 10.1186/1748-717X-2-4 (PMC1770926; doi:10.1186/1748-717X-2-4)

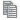

Supplement: Additional File 1 — Clono-Counter. Contains a manual for the program, the program itself and an example. [file 1748-717X-2-4-S1.zip › images/copy.gif]

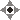

Supplement: Additional File 1 — Clono-Counter. Contains a manual for the program, the program itself and an example. [file 1748-717X-2-4-S1.zip › images/gt.gif]

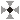

Supplement: Additional File 1 — Clono-Counter. Contains a manual for the program, the program itself and an example. [file 1748-717X-2-4-S1.zip › images/ls.gif]

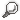

Supplement: Additional File 1 — Clono-Counter. Contains a manual for the program, the program itself and an example. [file 1748-717X-2-4-S1.zip › images/lupe.gif]

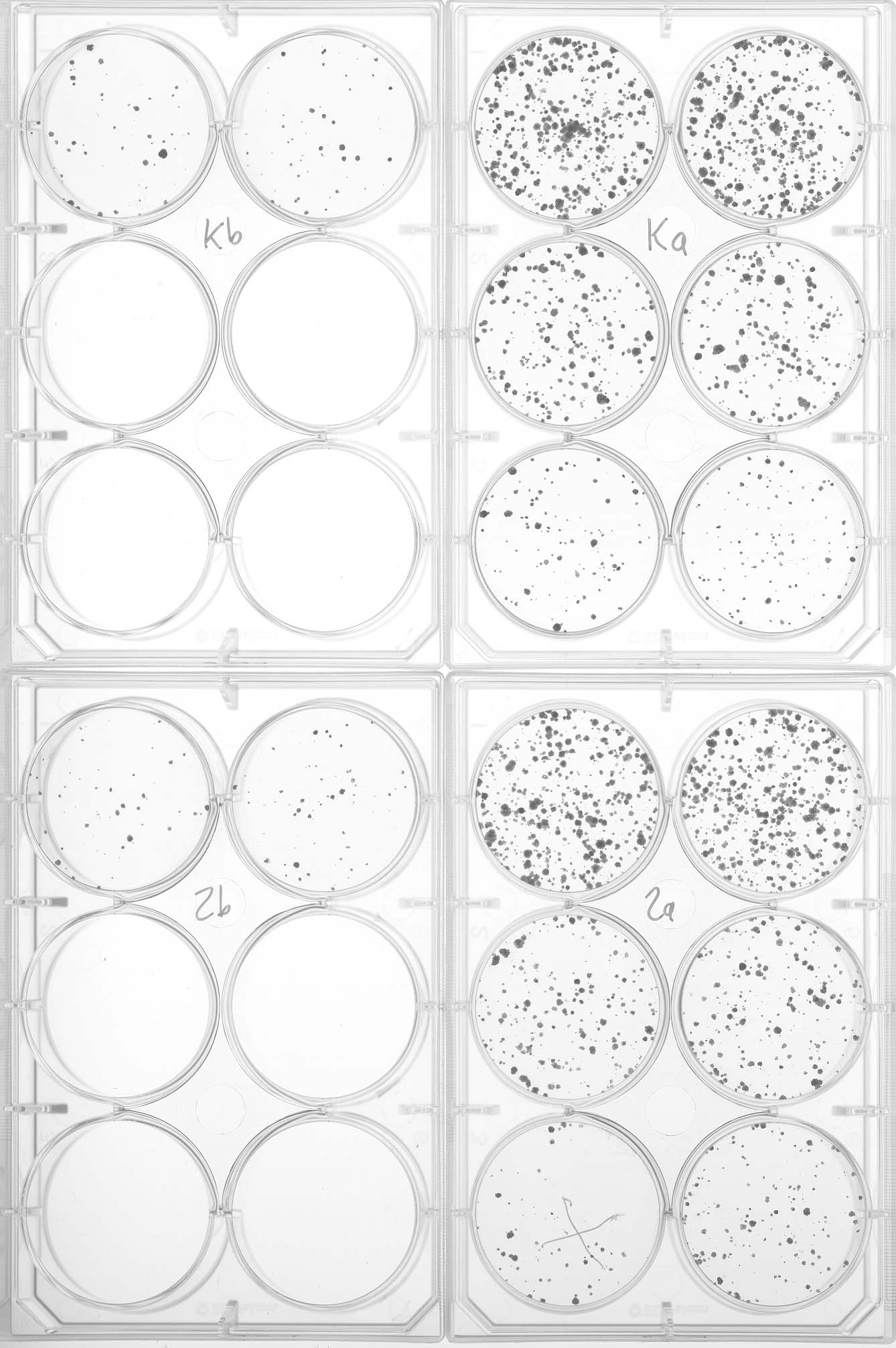

Supplement: Additional File 1 — Clono-Counter. Contains a manual for the program, the program itself and an example. [file 1748-717X-2-4-S1.zip › example.jpeg]
